# Genome Characterization of Mammalian Orthoreovirus and Porcine Epidemic Diarrhea Virus Isolated from the Same Fattening Pig

**DOI:** 10.3390/ani15020156

**Published:** 2025-01-09

**Authors:** Xiaoxuan Li, Jiakai Zhao, Jingjie Li, Yangzong Xiri, Zhixiang Liu, Qin Zhao, Yani Sun

**Affiliations:** Department of Preventive Veterinary Medicine, College of Veterinary Medicine, Northwest A&F University, Yangling 712100, China; lixiaoxuan10112@163.com (X.L.); jiakaizhao3111@163.com (J.Z.); 15351227807@163.com (J.L.); xx03020710@163.com (Y.X.); liuzhixiang968@163.com (Z.L.); qinzhao_2004@nwsuaf.edu.cn (Q.Z.)

**Keywords:** mammalian orthoreovirus, porcine epidemic diarrhea virus, genome, reassortment

## Abstract

In 2020, severe diarrhea occurred in four-month-old fattening pigs from nine farms in Shandong Province, China. Fecal samples were collected from diseased pigs and tested by PCR for the presence of mammalian orthoreovirus (MRV), porcine epidemic diarrhea virus (PEDV), porcine deltacoronavirus (PDCoV), porcine rotavirus A (PoRVA), transmissible gastroenteritis virus (TGEV), porcine kobuvirus (PKV), and pseudorabies virus (PRV). The viral RNA of MRV and PEDV was detected in the fecal samples. The genome sequences of MRV and PEDV were successfully amplified from the same fecal sample. Genomic and phylogenetic analysis showed that the MRV isolate named MRV2-SD/2020 belongs to serotype 2 MRV (MRV2) and may originate from the reassortment of human and porcine MRVs. Compared with other MRV2 strains, there were four other unique amino acid mutations (L274I, F302L, V347I, and T440M) in the receptor binding region. For the PEDV isolate named PEDV-SD/2020, the nearly complete genome was amplified from the positive fecal samples. Phylogenetic analysis showed that it was classified into the G2a genotype. Compared with CV777 and other PEDV variant strains, its spike (S) protein exhibited two unique mutations (S663T and L966M). This study first reports the co-infection of PEDV and MRV2 in the pigs and provides a new direction for the prevention and control of the diarrhea diseases.

## Simple Summary

Mammalian orthoreovirus (MRV) and porcine epidemic diarrhea virus (PEDV) are two causative agents of pig diarrhea and contagious intestinal disease. Generally, PEDV infection causes severe diarrhea, dehydration, and even death in piglets, and MRV causes mild gastroenteritis and respiratory diseases. In this study, the co-infection of MRV2 and PEDV was first determined in fattening pigs with diarrheal diseases and the two viral genome sequences were obtained from the same pig. The phylogenetic analysis based on the complete genome showed that the MRV2 strain was a reassortment of human and porcine MRV, and the PEDV strain belonged to the G2a genotype. This will help to better understand PEDV and MRV and provide a scientific basis for the development of effective prevention and control strategies.

## 1. Introduction

In recent years, the pig industry in China has witnessed rapid development and China has become the world’s largest pig producer. Nevertheless, during the process of pig production, the phenomena of “new diseases emerging suddenly and old diseases recurring” persist constantly, and complex and variable situations such as mixed and cross-infections of different pathogens keep recurring in pig farms. In particular, diarrhea is a prevalent condition in swine, and the associated dehydration is a leading cause of mortality, resulting in significant economic losses for the pig industry. The etiological agents of diarrhea are diverse, encompassing various viral, bacterial, and parasitic pathogens, with viruses being the predominant causative factor. The most common pathogens of viral diarrhea include a variety of viruses, such as porcine epidemic diarrhea virus (PEDV), porcine deltacoronavirus (PDCoV), and transmissible gastroenteritis virus (TGEV) co-infection, causing huge economic losses to the pig industry and also resulting in difficulties in the diagnosis and treatment of the disease [1,2].

PEDV is the most common diarrheal virus and has caused great concern in Asian countries such as China, South Korea, and Vietnam [3,4,5]. The prevalence of PEDV decreased in China from 2018 to 2020, which may be due to the widespread use of the PEDV vaccine (G2b) in China [6,7]. However, the detection rate of PEDV increased sharply in 2021.In particular, the co-infection with porcine rotavirus A (PoRVA) became popular in recent years, which will undoubtedly increase the pressure on PEDV prevention and control [8]. The co-infection rate of PEDV with one other virus was 66.97%, and the infection rate of PEDV with two or more other pathogens was 29.05% [9]. Recent studies have found that pigs can be co-infected with PEDV and MRV3 (MRV-ZJ2013), resulting in severe diarrhea and other clinical symptoms in piglets [10]. Therefore, the investigation of PEDV co-infection with other pathogens needs to be carried out continuously.

Shandong Province is one of the regions with the largest pig populations in China. Since 2010, severe diarrheal diseases have frequently occurred in the region and caused serious economic losses [11]. An epidemiological survey showed that PED was moderately highly prevalent in some areas of Shandong Province, and the positive rate of PEDV infection was up to 36.36% [12]. Since diarrhea caused by porcine enteric coronaviruses can cause similar clinical symptoms and pathological changes in pigs and is difficult to distinguish, singleplex and multiplex RT-PCR assays have been widely used for the molecular detection of porcine diarrhea viruses [13,14]. However, compared with traditional methods, next-generation sequencing (NGS) technology has the characteristic of high resolution and has become an important tool for pathogen screening and genetic background study [15]. Therefore, in the present study, to identify the etiology of acute diarrhea in pig farms, fecal samples were collected from diseased pigs and PEDV and MRV2 genomes were obtained by traditional PCR and NGS, respectively, which helped to better understand PEDV and MRV co-infection and provide a scientific basis for the development of effective prevention and control strategies.

## 2. Materials and Methods

### 2.1. Samples

**Figure 1:**
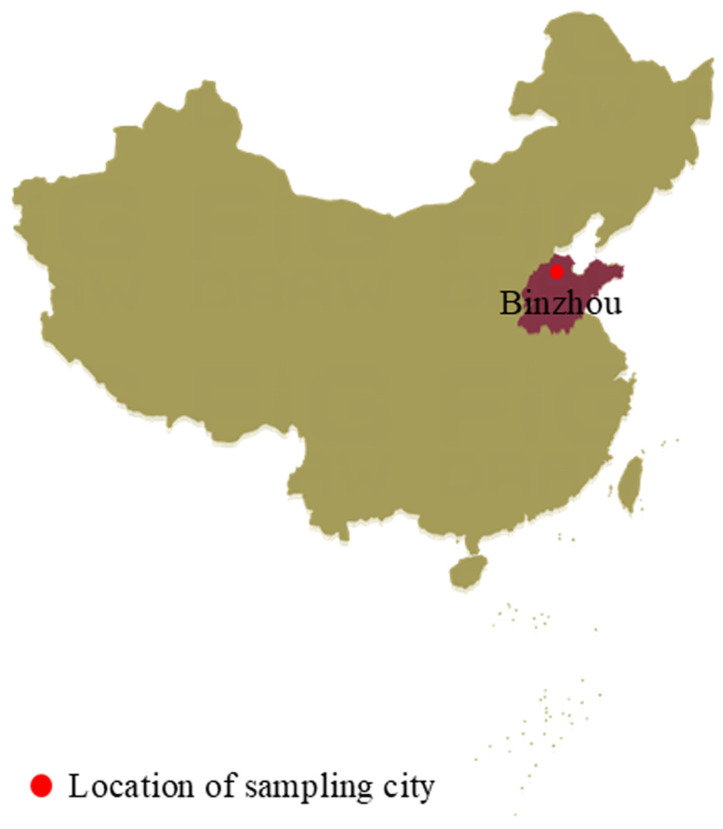
Map of sample collection in Binzhou City, Shandong Province, China.

In 2020, acute diarrhea occurred in 9 pig farms in Binzhou City (37°25′52.1″ N, 118°0′49.0″ E), Shandong Province, China. Each farm raised ~2000 fattening pigs (Figure 1). The diseased pigs showed anorexia, depression of the sensorium, watery diarrhea, and varying degrees of weight loss and dehydration. Necropsy showed that the intestinal wall was thinned and translucent, with yellow liquid inside the intestine. The morbidity and mortality were 63% and 3%, respectively. Next, a total of 93 fresh watery fecal samples were collected from the diseased pigs with watery diarrhea and submitted to our laboratory for the identification of etiologic agent(s).

### 2.2. Cell

Vero cells were purchased from the American Type Culture Collection and cultured in Dulbecco’s modified Eagle’s medium (DMEM; Gibco; Thermo Fisher Scientific, Waltham, MA, USA), supplemented with 10% fetal bovine serum (FBS; Gibco; Thermo Fisher Scientific, Waltham, MA, USA) in a humidified incubator at 37 °C with 5% CO_2_.

### 2.3. Detection of Viral DNA/RNA from the Samples

To determine the causative agent, the main viruses causing diarrhea in pigs, including PDCoV, PoRVA, TGEV, porcine kobuvirus (PKV), pseudorabies virus (PRV), MRV, and PEDV, were detected. The fecal samples were first homogenized in 0.1 M phosphate buffer (PBS) and centrifuged at 3500× *g* for 15 min. The supernatants were then collected for DNA/RNA extraction and virus isolation. Total DNA/RNA was extracted from the supernatants using EasyPure Viral DNA/RNA Kit (TransGen Biotech, Beijing, China), according to the manufacturer’s instructions. Primer information is shown in Table 1. The complementary DNA (cDNA) synthesis of six viruses, namely, PDCoV, MRV, PEDV, TGEV, PoRVA, and PKV, was performed by Prime Script RT Reagent Kit (TaKaRa, Tokyo, Japan). At the same time, the extracted DNA was used as a template for PRV detection. PCR was performed using 2 μL cDNA as template with added 2 x Taq PCR Master Mixture (TaKaRa, Tokyo, Japan) and thermal cycling using the following conditions: initial denaturation at 94 °C for 10 min; 35 cycles of denaturation at 94 °C for 30 s, annealing at 55 °C for 30 s, and extension at 72 °C for 2 min; with a final incubation of 72 °C for 5 min. The PCR products were analyzed with 1% acrylamide gel, followed by silver staining.

### 2.4. Virus Isolation

The supernatant was filtered through a 0.22-μm Millipore filter (Billerica, MA, USA) and subsequently inoculated into Vero cells for virus isolation. Briefly, the Vero cells were seeded into 6-well plates, and cultured with DMEM (Gibco; Thermo Fisher Scientific, Waltham, MA, USA) supplemented with 10% FBS (Gibco; Thermo Fisher Scientific, Waltham, MA, USA) and 1% antibiotics (penicillin and streptomycin, *w*/*v*) at 37 °C with 5% CO_2_. When the cells reached 70% confluence, 50 µL of the supernatant was added into the cells supplemented with 8 µg/mL of trypsin (Gibco; Thermo Fisher Scientific, Waltham, MA, USA) and incubated for 1.5 h at 37 °C under 5% CO_2_. The inoculum was then removed and the cells were washed twice with PBS. Subsequently, 2 mL of maintenance medium (DMEM; Gibco; Thermo Fisher Scientific, Waltham, MA, USA) without FBS supplemented with 4 µg/mL trypsin was added to each well. When the cytopathic effect (CPE) was observed in ~80% of the inoculated cells, the cells and supernatant were collected and freeze-thawed three times. Following centrifugation at 5000× *g* for 10 min at 4 °C, the supernatants were collected and stored at −80 °C. After five blind passages, the virus was purified by plaque assay and the virus titer was determined, as previously described [22]. The virus titer was calculated as the 50% tissue culture infectious dose (TCID_50_) using the Reed–Muench method [23].

### 2.5. Electron Microscopy Assay

Vero cells were infected with the isolated virus for 24 h at a 1 MOI and the cells were then fixed with 2.5% glutaraldehyde in 0.1 M sodium phosphate buffer (pH 7.4) for 12 h at 4 °C. The cells were fixed with 2% osmium tetroxide for 3 h and dehydrated in a graded series of ethanol dilutions (30, 50, 70, 80, 90, 95, and 100%) for 20 min at each step. The cells were then placed in one of four mixtures of anhydrous ethanol and LR-White resin (3:1, 1:1, 1:3, and pure LR-White resin) for 2, 8, 12, and 24 h, respectively. Finally, the ultrathin sections were stained with uranyl acetate and lead citrate for 5–10 min and observed using a FEI Tecnai G2 Spirit transmission electron microscope (Hillsboro, OR, USA).

### 2.6. Amplification of the Complete Genome

The complete genome of the isolated MRV2 virus was sequenced by NovaSeq 6000 Illumina. Briefly, total RNA was extracted from the supernatants of virus-infected cells using EasyPure Viral DNA/RNA Kit (TransGen Biotech, Beijing, China). According to the manufacturer’s instructions, the random amplicon was placed into the Nextera XT DNA Library Preparation Kit (Illumina, San Diego, CA, USA) and the genome was sequenced using NovaSeq 6000 (Illumina, San Diego, CA, USA). Raw reads were filtered and trimmed by fastp (https://github.com/OpenGene/fastp (accessed on 25 January 2024)) to remove sequencing adapters and low-quality reads, including those reads scoring <Q20. Ribosomal RNAs and host read subtraction by read-mapping were performed using the BBMap tool. De novo genome assembly was performed using SPAdes v3.13.0. These extracted assembled scaffolds limited the minimum contig length to 100 bases, with the best BLAST hits to the NCBI nucleotide (nt) database. Finally, the complete genome was obtained and annotated based on those annotations of genomic sequences in the SwissProt database and then submitted to GenBank.

In addition, since we did not isolate PEDV from the fecal samples, the PEDV whole genome was obtained by Sanger sequencing and the 12 fragments of the PEDV genome were amplified from the total RNA of fecal samples by the classical RT-PCR. For amplifying the genome of PEDV in the fecal samples, 12 pairs of specific primers (Table 2) were designed based on the sequences of PEDV IA1 (GenBank No. KF468753.1). Total RNA was also directly extracted from the fecal samples using TRIzol^®^ reagent, according to the manufacturer’s instructions (TaKaRa, Tokyo, Japan). Reverse transcription was performed using MMLV reverse transcriptase (TaKaRa, Tokyo, Japan) under the following conditions: 42 °C for 60 min and 85 °C for 15 min. PCR was performed using 1 µL cDNA as template with added PrimeSTAR GXL DNA Polymerase (TaKaRa, Tokyo, Japan) and thermal cycling using the following conditions: 98 °C for 5 min, 30 cycles of 98 °C for 10 s, 55 °C for 30 s, and 68 °C for 2 min, with a final incubation at 68 °C for 10 min. The PCR products were purified by gel extracted and sequenced by Beijing Qingke Biotechnology Co., Ltd. The near-complete genomic sequences were edited and assembled using the EditSeq program (version 7.1.0) of the Lasergene software package (version 7.1.0) (DNASTAR, Madison, WI, USA). All the sequences were determined from at least three independent PCR products.

### 2.7. Sequencing and Phylogenetic Analysis

The complete genomes obtained in the present study were compared with reference sequences downloaded from GenBank using MegAlign program 7.1.0 of the Lasergene software package (Madison, WI, USA). The MEGA 7.0 program was used to perform multiple sequence alignment and to subsequently build maximum likelihood phylogenetic trees with bootstrap support [24].

## 3. Results

### 3.1. Virus Isolation and Identification

**Figure 2:**
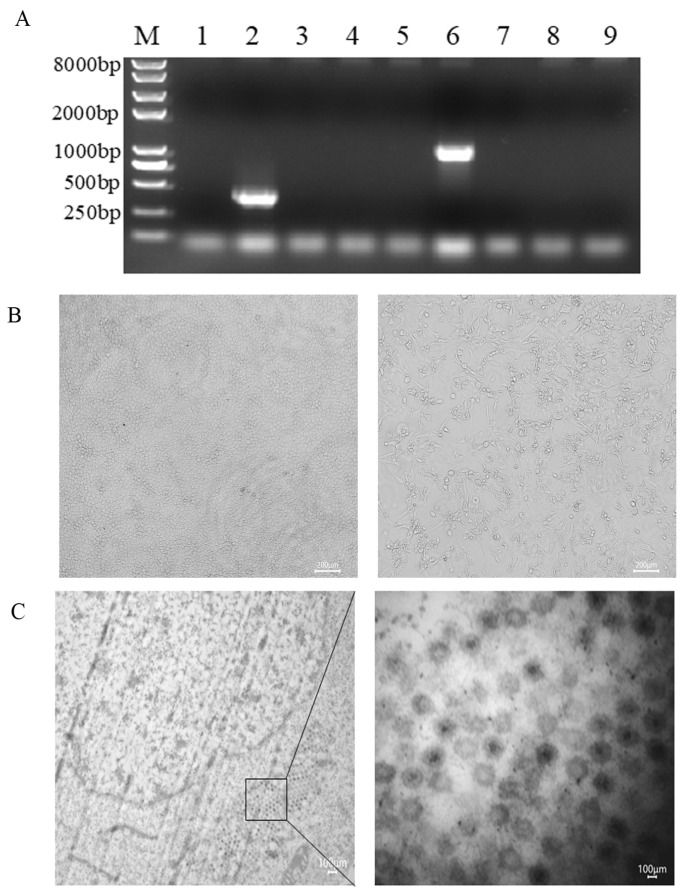
Isolation and identification of PEDV and MRV. (**A**) Fecal samples were used as a template for the RT-PCR detection of the viruses (Lane M: DL 8000 bp marker; Lane 1, classical strains of PEDV; Lane 2, variant strains of PEDV; Lane 3, PDCoV, Lane 4, TGEV; Lane 5, PoRVA; Lane 6, MRV; Lane 7, PKV; Lane 8, PRV; Lane 9, negative control). (**B**) MRV-infected Vero cells at 2 dpi became round and enlarged, and then detached. Mock-infected Vero cell cultures showing normal cells. (**C**) Electron microscopy of negatively stained virus-infected Vero cells showed a single cell infected with MRV2-SD/2020.

To determine the causative agent of diarrhea in the fattening pigs, total DNA/RNA was extracted from the fecal samples and used as the template to detect the main virus DNA/RNA by PCR/RT-PCR. The results showed that the expected size bands of PEDV and MRV were successfully amplified, while the ones of PDCoV, PoRVA, TGEV, PKV, and PRV were all negative (Figure 2A). Of those, 73 were positive for PEDV RNA, 61 were positive for MRV RNA, and 57 were positive for both viral RNAs. These results suggested that the pigs may be co-infected with PEDV and MRV.

The positive samples for PEDV and MRV RNA were processed and used for virus isolation on Vero cells, as previously described [22,25]. The RNA of the continuously propagated virus was extracted and identified by RT-PCR. The results showed that the RNA was negative for the PEDV S gene after three passages. However, it was positive for the MRV S1 gene after three passages. At 2 days post-infection (dpi), the infected Vero cells became round and detached, as compared with the negative control (Figure 2B). Following plaque purification and identification, an MRV strain was successfully isolated with the Vero cells. The isolate was then named MRV2-SD/2020 with a viral load of 10^5.75^TCID_50_/0.1 ml. The electron microscopy of the infected Vero cells showed icosahedral non-enveloped virus particles neatly arranged in the cytoplasm of infected cells, ranging in size from 70 to 90 nm (Figure 2C). These results suggested that an MRV strain was successfully isolated, but PEDV was not isolated.

### 3.2. Sequence Analysis of MRV Isolate

MRV2 was subjected to NGS analysis using the NovaSeq 6000 platform. The viral genome targeted assembly pipeline (Virus TAP) analysis covered the 10 segments of the MRV genome (length 3803, 3869, 3827, 2211, 2126, 2186, 1383, 1256, 1166, and 1097 bp), which encode 10 open reading frames (L1, L2, L3, M1, M2, M3, S1, S2, S3, and S4). The 10 segments of the MRV2-SD/2020 have been uploaded to GenBank under the accession numbers PP951610 to PP951619. Pair-wise nt and aa comparisons between MRV2-SD/2020 and other orthoreoviruses, including the prototype MRVs, were performed for all 10 segments (Table 3). The S1 gene encodes σ1 protein, which is the serotype-specific antigen of MRV [26]. The homology comparative analysis of S1 showed that MRV2-SD/2020 had the highest homology with the 117 belonging to the MRV2 group, with nt and aa homology of 90.6% and 91.5%, respectively. However, the homologies of nt with other serotype MRVs were only 26–56%. The results indicated that the MRV2-SD/2020 isolate belonged to MRV2. The nt and aa homology of L2 was close to that of MRV1 with an identity of 95.08% and 97.8%, respectively. The nt and aa homology of M2 was close to that of MRV3 with identity of 97% and 98.6%, respectively. Besides S1, L2, and M2, comparative analysis showed that the remaining seven segments had the highest homology with the other MRV2 strains and shared nt and amino acid (aa) homology from 92.32% to 98% and from 88.4% to 99.2% (Table 3). Subsequently, the amino acid sequences of the MRV2-SD/2020 S1 protein were aligned with the other S1 proteins of MRV2 strains. The results revealed nine unique amino acid mutations (N73D, I82V, N191D, Q200K, G218S, L274I, F302L, V347I, and T440M) (Table 4). Among them, four unique amino acid mutations (L274I, F302L, V347I, and T440M) were in its receptor binding region (Table 4).

**Figure 3:**
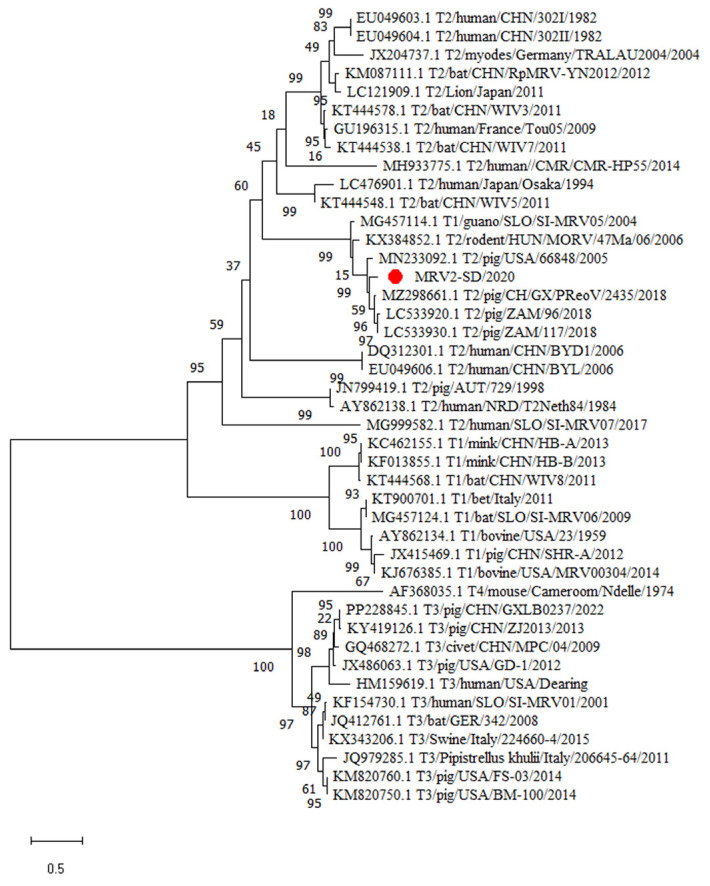
Phylogenetic analysis of the S1 genome segments for the MRV2-SD/2020 strain. Phylogenetic analyses were performed using the maximum likelihood method using MEGA 7.0 software. The strain isolated in this study is indicated with a red circle (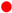).

To establish the evolutionary relationship between MRV2-SD/2020 and other known orthoreoviruses, 10 phylogenetic trees were separately constructed based on the nt sequence per 10 segments using the maximum likelihood method (Figure 3 and Figures S1–S3). The phylogenetic tree showed that the S1 segment of MRV2-SD/2020 was closely associated with porcine MRV2 isolates (CH/GX/PReoV/2435/2018, 96 RNA and 117) from China and Japan, further indicating that the MRV2-SD/2020 was a serotype 2 strain. In addition, the phylogenetic trees based on the L1, L2, L3, M1, M2, M3, S2, and S4 segments showed that the different segments of MRV2-SD/2020 were separately clustered with different porcine strains. Of note, the phylogenetic tree based on the S3 segment showed that the one of MRV2-SD/2020 was closely associated with the MRV Osaka1994 strain from a human. These results suggested that the MRV2-SD/2020 isolate was also a reassortment strain similar to other orthoreovirus isolates, and that the S3 segment may be from a human isolate.

### 3.3. Sequence Analysis of PEDV Isolate

The nearly complete genome of PEDV was also amplified from the fecal samples. The genomic sequence was with the size of 27,996 nt, excluding the poly A tail, and was named PEDV-SD/2020 (GenBank accession no. PP958821). Next, the complete genome of PEDV-SD/2020 was used for multiple alignment and phylogenetic analysis with PEDV sequences in GenBank. The alignment showed that PEDV-SD/2020 exhibited 96.4–97.4% nt identities with the reference G1 strains, 98.6–99% nt identities with the reference G2a strains, 97.7–98.9% nt identities with the reference G2b strains, and 98.5–98.7% nt identities with the reference S-INDEL (insertions and deletions) strains (Table 5). The results indicated that the PEDV-SD/2020 strain belongs to the G2a group. Compared with the G2a strain, PEDV-SD/2020 had more amino acid mutations in the complete genome sequence, including 24 mutations in the ORF1a and ORF1b gene, 3 mutations in the S gene, and no mutations in other genes. These mutations, especially those in the S protein, may be its pathogenic determinants, because the S protein contains many epitopes and is highly variable, and amino acid substitutions, deletions, or insertions can significantly change the pathogenicity and antigenicity of the virus [27,28]. The amino acid and nt homology analysis of the PEDV S gene showed that the PEDV-SD/2020 gene has the highest nt and amino acid homology with the IA1-USA strain (98.8% and 99.1%, respectively) and the lowest homology with the LZC/China strain (93.4% and 92.6%, respectively) (Table S1).

**Figure 4:**
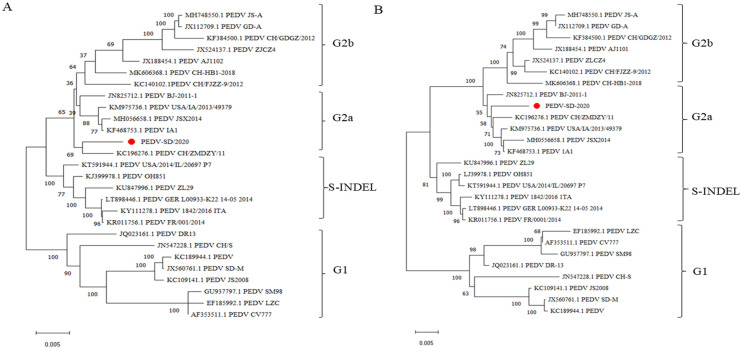
Phylogenetic analysis of the PEDV complete genome and S genes. (**A**) Multiple sequence alignments were generated with Clustal X, and a complete genome phylogenetic tree was constructed using the maximum likelihood method in the MEGA 7.0 software. (**B**) The S gene phylogenetic tree was constructed using the same method as above. The strain isolated in this study is indicated with a red circle (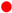).

To date, six neutralization epitopes for PEDV S proteins have been determined, namely, S1^0^ (19–220 aa), S1^A^ (aa 435–485), core center epitopes (499–638 aa), SS2 (748–755 aa), and SS6 (764–771 aa) in the S1D region, and the C-terminus epitope 2C10 (1368–1374 aa) [29,30]. Compared with the CV777 strain, the PEDV-SD/2020 S protein has nine amino acid substitutions (A517S, L521H, S523G, V527I, T549S, G594S, A605E, L612F, and I635V) in the core neutralizing epitope (COE) and one amino acid substitution (Y766S) in SS6. In addition, the PEDV-SD/2020 S protein had two unique mutations (S663T and L966M) that were not present in other PEDV variants and CV777 strains (Table 6). Phylogenetic analysis based on the complete genome and S protein showed that PEDV-SD/2020 was closely associated with the WHLL, GD-B, BJ-2011-1,1A1, and PEDV-CHZ strains that were G2a variants, and was distinct from the classical strain CV777 (Figure 4A,B).

## 4. Discussion

As a major country in the world’s pig industry, China has faced serious economic losses due to viral diarrheal diseases in pigs caused by a variety of pig intestinal pathogens. In recent years, the co-infection of porcine enteric coronaviruses has become common in natural infections. In addition, the co-infection of PEDV and MRV3 in Chinese pig was reported in 2017, which led to the aggravation of the disease in piglets [10]. It was documented that MRV3 (MRV-ZJ2013) was a low-pathogenic strain in piglets, but when the animals were co-infected with PEDV, this could lead to severe diarrhea and other clinical symptoms in piglets, which aggravated PEDV infection [10]. In the present study, a co-infection of PEDV and MRV2 was first characterized from diarrheic fattening pigs in Binzhou City of Shandong Province. Based on the clinical signs of the diseased pigs, it was also suspected that MRV2 and PEDV co-infection was the main reason for the aggravation of the diarrheal disease in Binzhou City. As we know, MRV could infect humans and pigs, which means that the swineherd is at risk of infection and genome variation or virulence increasing. To be sure, more epidemiology investigations are needed to be assessed to understand the epidemic situation of PEDV and MRV2 co-infection in China.

Because most enteroviruses cause similar clinical manifestations in pigs, it is important to quickly differentiate the specific pathogen causing pig diarrhea in order to control the disease. Compared with traditional sequencing, NGS can rapidly identify the unknown pathogens in a short time. Therefore, we determined the pathogen and performed whole-genome sequencing of MRV2-SD/2020 using the NGS method. The dsRNA genome of MRV contains 10 discrete segments, which are susceptible to various types of alterations, including intragenic rearrangements and reassortment. The virulence and biological properties of the recombined virus are unpredictable, and may even lead to the creation of new pathogenic virus strains through recombination [10]. In recent years, the infection rate of MRV has significantly increased in China. In 2017, Ye et al. isolated a recombinant in the L3 gene of MRV from diarrheic piglets in Heilongjiang [31], and Yan et al. isolated a recombinant MRV strain with human, deer, cattle, and civet gene segment from bats in Xinjiang [32]. Due to the apparent lack of species barrier, the potential for MRV to spread from animals to humans makes it an important zoonotic pathogen. In this study, the homology and genetic evolutionary relationship of each gene segment indicated that, except for S1 and L2, the remaining eight segments had the highest homology with the other MRV strains and shared nt and aa homology from 95.08% to 98% and from 97.8% to 99.2%. This is consistent with the higher variability of MRV S1 protein reported by other studies. In addition, the 10 fragments had the highest homology with the isolates from China and Japan, and we speculated that the prevalence of MRV was closely related to the region. It is worth noting that the S3 segment was related to MRV2 Osaka isolated from humans, which was first isolated from a child with meningitis in 1994, while the other segments were related to pig MRV [33]. Therefore, we speculated that the MRV2-SD/2020 strain was a human–pig reassortment virus. However, whether this strain can spread between humans and pigs remains to be further studied. Although the protein functions of various segments have been predicted, apart from the S1 segment, few studies have been reported for other segments. Furthermore, it is known that the S1 segment is unique to each MRV strain and determines the serotype [26]. Based on the phylogenetic analysis of the S1 segment, MRV2-SD/2020 was closely associated with MRV-117/Japan (GenBank No. LC533930) and was classified into MRV2 in this study. To date, MRV2 has been identified and isolated in several countries, such as Italy, the United States, and Japan [34,35]. It was not until 2018 that MRV2 was isolated for the first time from piglets with diarrhea in China, and it has so far not caused a widespread epidemic [22]. The σ1 protein has an independent host cell binding ability, and is mainly responsible for cell attachment, tissue tropism, and hemagglutination [26]. According to the amino acid sequence analysis of the σ1 protein, MRV2-SD/2020 had four other unique amino acid mutations (L274I, F302L, V347I, and T440M) in the receptor binding region (256–468 aa). However, the biological significance of these amino acid sites remains unclear. Therefore, the effect of the unique amino acid changes in the σ1 protein of this MRV2 strain needs to be further investigated.

PEDV is a deadly intestinal disease for piglets worldwide. Since the large-scale outbreak of PEDV in Asia in 2010, the highly pathogenic variant (G2 subtype) has been the epidemic strain, and caused major economic losses in the breeding industry [36]. Zhuang et al. showed that the prevalence of PEDV G2 reached 92.3% in 17 provinces of China from 2020 to 2021 [37]. Chen et al. also showed that the PEDV G2 subtype strains were mainly present in Shandong from 2019 to 2021 [38]. In the present study, PEDV RNA was detected in the feces samples from diarrheic pigs in Shandong Province, China. Phylogenetic tree analysis based on the complete genome and S protein showed that the PEDV-SD/2020 strains belonged to the G2a genotype, which indicated that the G2a genotype may still be predominant in China. Compared with the G2a strain, PEDV-SD/2020 had more amino acid mutations in the ORF1a, ORF1b, and S genes. The PEDV ORF1a, ORF1b, and S genes have been reported to be involved in virus entry, proliferation, and synthesis [39,40]. In this study, after three blind passages in Vero cells, the virus could be isolated. It has been reported that the limited suitability of cells for PEDV culture, low proliferation potency, complex culture conditions, and other factors still seriously restrict PEDV isolation in vitro [41]. Hofmann et al. and Kusanagi et al. reported an adaptation to the serial propagation of PEDV in Vero cells by the addition of trypsin to the culture medium and the appearance of CPE characterized by cell fusion and syncytial formation [42,43]. The present study also referred to the previously reported method of PEDV isolation by trypsin, without success. It may be suggested that these amino acid mutations may affect the proliferation of MRV2-SD/2020 on Vero cells. So the mutations in the ORF1a, ORF1b, and S proteins require to be further analyzed. A detailed study of amino acid mutation in the ORF1 and S protein would also be useful for exploring efficient cell culture systems and methods for PEDV. Another possible reason is that after co-infection with MRV2, MRV2 proliferated rapidly in Vero cells and inhibited PEDV proliferation, resulting in the inability of PEDV to replicate.

As we know, the PEDV S protein plays a key role in the induction of neutralizing antibodies, which makes it become a primary target for the development of effective vaccines against PEDV [29,44]. Therefore, paying attention to the variation in the PEDV S protein may be more conducive to understanding the epidemic situation of PEDV. As previously reported, amino acid mutations in the COE and SS6 epitopes and large mutations at amino acid positions 713 to 916 of the S protein result in altered virulence and reduce the effectiveness of conventional vaccines, which may also explain why PEDV remains prevalent despite the availability of vaccines [45,46,47]. In this study, compared with the CV777 strain, the PEDV-SD/2020 S protein has nine amino acid substitutions in COE, one amino acid substitution in SS6, and two unique mutations (S663T and L966M) in the S protein. The deletion, insertion, and mutation of the S protein may affect the protein structure and indirectly influence the virulence of PEDV [29]. Therefore, it remains to be investigated whether the unique amino acid changes in the S protein of the PEDV-SD/2020 strain affect the antigenicity and pathogenicity of PEDV.

## 5. Conclusions

In this study, the co-infection of MRV2 and PEDV was first determined in fattening pigs with diarrheal diseases. The MRV2 strain was successfully isolated and the genomes of MRV2 and PEDV were characterized. The MRV2 strain was a reassortment strain of human and porcine MRV. And two mutation sites on the PEDV S protein were different from those of other PEDV strains.

## Figures and Tables

**Table 1:** Sequences of primers used in this study.

Primer	Sequence (5′–3′)	Size (bp)
PDCoV F	5′-TTTCAGGTGCTCAAAGCTCA-3′	447 [16]
PDCoV R	5′-GTTAACAGATTGAGATCTTGG-3′	
PoRVA-F	5′-GGCTTTAAAAGAGAGAAT-3′	1067 [17]
PoRVA-R	5′-GGTCACATCATACAATTC-3′	
PRV-F	5′-CCGTGTTCTTTGTGGCGGTG-3′	421 [18]
PRV-R	5′-ACCTCCTCGCCGAAGGCGTCGAAG-3′	
TGEV-F	5′-CTTGGTAGTGGTGCTA-3′	529 [19]
TGEV-R	5′-CTATCTGGTCGCCATCTTC-3′	
PEDV-F	5′-TTGCAAGTGGCGCTGTGATT-3′	
PEDV-R1	5′-GCCGACAACAATATCTTTTCCA-3′	747 [20]
PEDV-R2	5′-GACACCCTGGTTTTCACCAA-3′	442
PKV-F	5′-TGGACGACCAGCTCTTCCTTAAACAC-3′	495 [21]
PKV-R	5′-AGTGCAAGTGCAAGTCTGGGTTGCAGCC-3′	
MRV-S1-F	5′-GGGTCAACGCGTCGATGC-3′	1087
MRV-S1-R	5′-TCAACCGAGACAGGGATA-3′	

**Table 2:** Primers for whole genome sequencing.

Primer Pairs	Upstream Primer (5′–3′)	Downstream Primer (5′–3′)	Location, bp
PEDV-1	ATGTGGACACTTTTGGTA	ACTCAAAACCTGACATCGTG	513–2406
PEDV-2	ACATTACGGCCCATGAAC	AAGGAACACTAAAAATTC	2330–4538
PEDV-3	CAGAAGTGCTCAAATGAT	ATTACCAACTTCAATTGC	4322–7060
PEDV-4	GCCTTAAGCGCGTTCCTG	ACGTGTCGTTGTGATTAG	6885–9240
PEDV-5	TATTCAGGCAGTGCTTCA	GCCTTACCTTCTCCAACTATG	9150–11,947
PEDV-6	CTGAGTCCCTGTCATGGC	AAACTTAGTAGTACCAAT	11,808–14,340
PEDV-7	ATAAGTGGCAAAGAACGT	CATACGGCTGGCAACATA	14,217–16,950
PEDV-8	CAGATAGCAAGCAGTGTT	CCACATTTTACAATCGAC	16,770–19,673
PEDV-9	CTAGTAATGATAGCACGT	AGGTTTGTAGAGGAAATG	19,498–22,090
PEDV-10	ACGTTTCTGGTTTTTGGA	ATGCAGCAGAACACTAGT	21,933–24,678
PEDV-11	CAGAGTCTCTCCGTAATA	ATTCTGAATTACCGCG	24,498–26,905
PEDV-12	ACCCACTAACTTGGGTGT	TTTTTTTTTGTGTATCCA	26,690–28,043

**Table 3:** Highest nucleotide identities for each segment of the novel MRV2-SD/2020.

MRV2-SD/2020	Strain	Similarity (nt/aa) %	Serotype	Host	Country	GenBank
L1	CH/GX/PReoV/ 2435/2018	92.32/88.4	MRV-2	pig	CHN	MZ298655
L2	SHR-A	95.08/97.8	MRV-1	pig	CHN	JX415467
L3	96 RNA	97.83/99.2	MRV-2	pig	Japan	LC533916
M1	96 RNA	97/98	MRV-2	pig	Japan	LC533917
M2	GXLB0237	97/98.6	MRV-3	pig	CHN	PP228850
M3	117	98/98	MRV-2	pig	Japan	LC533929
S1	117	90.6/91.5	MRV-2	pig	Japan	LC533930
S2	117	97.96/99.1	MRV-2	pig	Japan	LC533931
S3	Osaka1994	97.73/99.2	MRV-2	human	Japan	LC476903
S4	117	96.81/98.2	MRV-2	pig	Japan	LC533933

**Table 4:** Amino acid sequence variations in the MRV S1 proteins.

Strains	Accession Number	Amino Acid Residues								
		73	82	191	200	218	274	302	347	440
302Ⅰ	EU049603	N	I	N	Q	G	L	F	V	T
302Ⅱ	EU049604	N	I	N	Q	G	L	F	V	T
TRALAU2004	JX204737	N	I	N	Q	R	L	F	V	T
RpMRV-YN2012	KM087111	N	I	N	Q	G	L	F	V	T
MRV-2	LC121909	N	I	N	Q	G	L	F	V	T
WIV7	KT444538	N	I	N	Q	G	L	F	V	T
Tou5	GU196315	N	I	N	Q	G	L	F	V	T
WIV3	KT444578	S	I	N	Q	G	L	F	V	T
CMR-HP	MH933775	N	I	N	R	G	L	F	V	T
Osala	LC476901	N	I	N	Q	G	L	F	V	T
WIV5	KT444548	N	I	N	Q	G	L	F	V	T
SI-MRV05	MG457114	N	I	N	Q	G	L	F	V	T
47Ma06	KX384852	N	I	N	Q	G	L	F	V	T
66848	MN233092	N	I	N	R	G	L	P	V	T
PReov/2435	MZ298661	N	I	N	Q	G	L	T	V	T
96	LC533920	N	I	N	Q	G	L	T	V	T
117	LC533930	N	I	N	Q	G	L	A	V	T
BYD1	DQ312301	N	I	N	Q	G	L	F	V	T
BYL	EU049606	N	I	N	Q	G	L	F	V	T
729	JN799419	N	I	N	Q	G	S	Y	V	T
T2Neth84	AY862138	N	I	N	Q	G	S	Y	V	T
SI-MRV07	MG999582	N	L	T	T	G	L	F	V	T
MRV2-SD/2020	PP951616	D	V	D	K	S	I	L	I	M

**Table 5:** Nucleotide sequence identity (%) of complete genome between PEDV-SD/2020 and different PEDV strains.

Strain Name	Countries	Genotype (%)	Time	Accession No.	Full Length
SM98	South Korea	G1 96.4	1998	GU937797.1	27,994
SD-M	China	G1 97	2012	JX560761.1	27,953
CH-S	China	G1 97.1	1986	JN547228.1	28,026
JS2008	China	G1 97.1	2008	KC109141.1	27,953
CV777	Switzerland	G1 96.6	1978	AF252511.1	28,033
KC189944.1	China	G1 96.9	2012	KC189944.1	27,953
LZC	China	G1 96.4	2006	EF185992.1	28,042
Virulent DR13	South Korea	G1 97.4	1999	JQ023161.1	28,029
JSX-2014	China	G2a 98.9	2014	MH056658.1	28,015
CH-ZMDZY-11	China	G2a 98.9	2011	KC196276.1	28,038
CH-FJZZ-9-2012	China	G2a 98.6	2012	KC140102.1	28,038
BJ-2011	China	G2a 98.9	2011	JN825712.1	28,038
IA1	USA	G2a 99	2013	KF468753.1	28,038
GD-A	China	G2b 98	2012	JX112709.1	28,035
USA-IA-2013-49379	USA	G2b 98.9	2013	KM975736.1	28,038
HBQHD1	China	G2b 98.7	2018	MK606368.1	28,038
CH-GDGZ-9-2012	China	G2b 97.7	2013	KF384500.1	28,035
AJ1102	China	G2b 98.4	2011	JX188454.1	28,044
JS-A	China	G2b 97.9	2019	MH748550.1	28,045
ZJCZ4	China	G2b 98	2011	JX524137.1	28,038
IT-A	Italy	S-INDEL 98.5	2017	KY111278.1	28,041
OH851	USA	S-INDEL 98.7	2014	KJ399978.1	28,029
GER-L00933-K22	Germany	S-INDEL 98.7	2018	LT898446.1	28,029
USA-2014IL-20697	USA	S-INDEL 98.6	2017	KT591944.1	28,025
FR-001-2014	France	S-INDEL 98.7	2014	KR011756.1	28,024
ZL29	China	S-INDEL 98.6	2016	KU847996.1	27,989

**Table 6:** Amino acid sequence variations in the PEDV-SD/2020 S proteins.

Strains	Accession Number	Amino Acid Residues												Genotype
		517	521	523	527	549	594	605	612	635	663	766	966	
GD-A	JX112709.1	A	H	G	I	S	S	E	F	V	S	S	L	G2b
GDGZ	KF384500	A	H	G	I	S	S	E	F	V	S	S	L	G2b
AJ1102	JX188454.1	A	H	G	I	S	S	E	F	V	S	S	L	G2b
ZJCZ4	JX524137.1	A	H	G	I	S	S	E	F	V	S	S	L	G2b
HBQHD1	MK606368.1	S	S	G	I	S	S	E	F	V	S	S	L	G2b
IA	KM975736.1	S	H	G	I	S	S	E	F	V	S	S	L	G2b
JS-A	MH748550.1	A	H	G	I	S	S	E	F	V	S	S	L	G2b
FJZZ	KC140102.1	A	H	G	I	S	S	E	F	V	S	S	L	G2a
JSX2014	MH056658.1	S	H	G	I	S	S	E	F	V	S	S	L	G2a
ZMDZY	KC196276.1	S	R	G	T	S	S	E	F	V	S	S	L	G2a
IAI	KF468753.1	S	H	G	I	S	S	E	F	V	S	S	L	G2a
BJ-2011	JN825712.1	S	L	G	I	S	S	E	F	V	S	S	L	G2a
CV777	AF252511.1	A	L	S	V	T	G	A	L	I	S	Y	L	G1
PEDV-SD/2020	PP958821	S	H	G	I	S	S	E	F	V	T	S	M	G2a

## Data Availability

The data supporting this study’s findings are available on request from the corresponding authors.

## Supplementary Materials

The following supporting information can be downloaded at: https://www.mdpi.com/article/10.3390/ani15020156/s1, Figure S1: Phylogenetic analysis of the L1–L3 genome segments for the MRV2-SD/2020 strain. (A) L1 segment. (B) L2 segment. (C) L3 segment. The strain isolated in this study is labeled by a red circle (). Figure S2: Phylogenetic analysis of the M1–M3 genome segments for the MRV2-SD/2020 strain. (A) M1 segment. (B) M2 segment. (C) M3 segment. The strain isolated in this study is labeled by a red circle (). Figure S3: Phylogenetic analysis of the S2–S4 genome segments for the MRV2-SD/2020 strain. (A) S2 segment. (B) S3 segment. (C) S4 segment. The strain isolated in this study is labeled by a red circle (). Table S1: Nucleotide and amino acid homology of S proteins of PEDV/SD/2020 isolates.
