# Supplementary material for: Genome Characterization of Mammalian Orthoreovirus and Porcine Epidemic Diarrhea Virus Isolated from the Same Fattening Pig
Source: Animals (Basel). 2025 Jan 9;15(2):156. doi: 10.3390/ani15020156 (PMC11758326; doi:10.3390/ani15020156)
Supplement: Supplementary file 1 [file animals-15-00156-s001.zip › animals-3377154-supplementary.pdf]

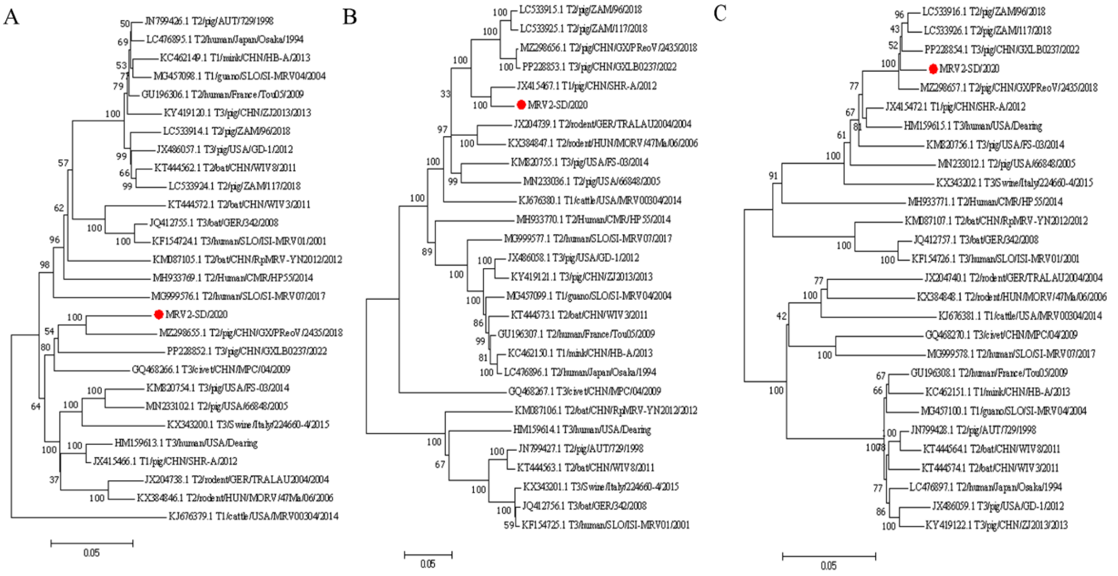

Figure. S1 Phylogenetic analysis of the L1-L3 genome segments for the MRV2-SD/2020 strain. (**A**) L1 segment. (**B**) L2 segment. (**C**) L3 segment. The strain isolated in this study is labeled by a circle ●.

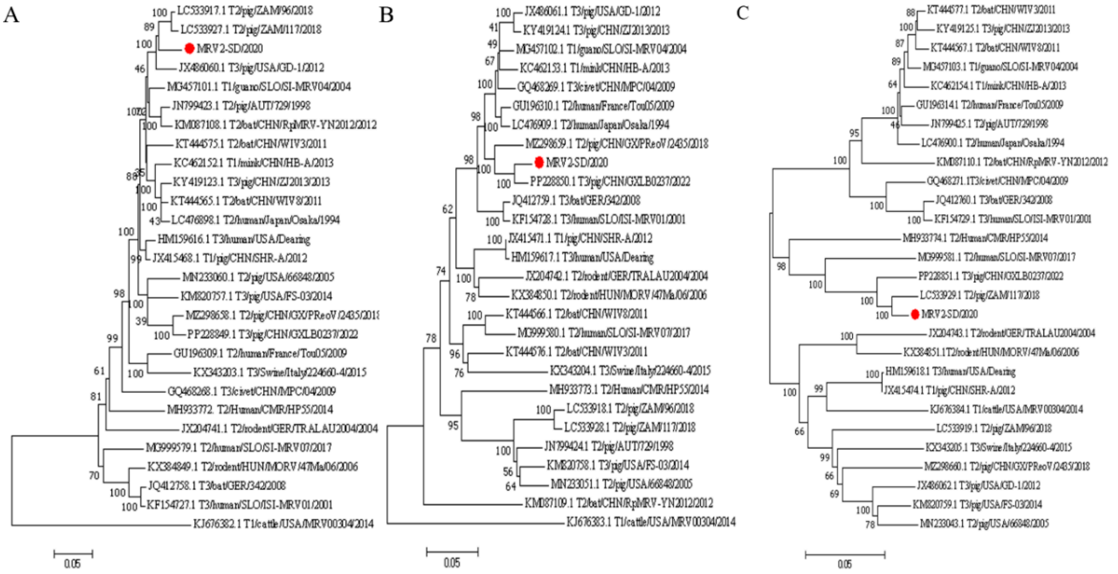

Figure. S2 Phylogenetic analysis of the M1-M3 genome segments for the MRV2-SD/2020 strain. (**A**) M1 segment. (**B**) M2 segment. (**C**) M3 segment. The strain isolated in this study is labeled by a circle ●.

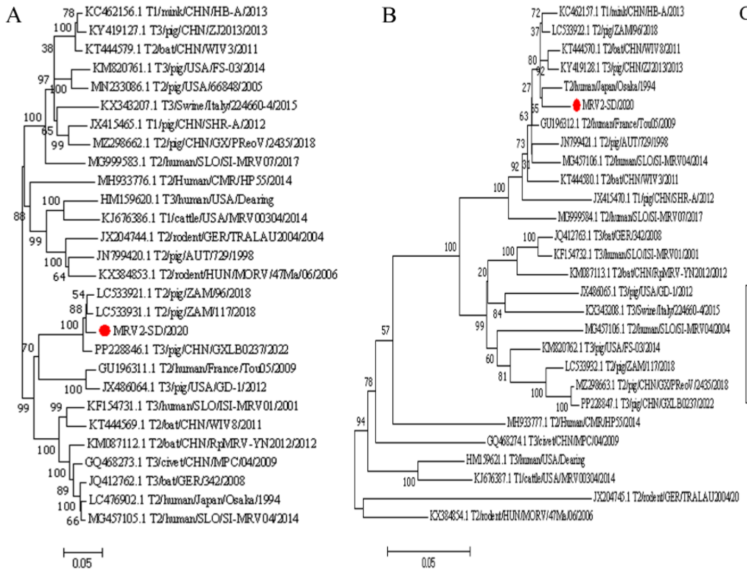

Figure. S3 Phylogenetic analysis of the S2-S4 genome segments for the MRV2-SD/2020 strain. (**A**) S2 segment. (**B**) S3 segment. (**C**) S4 segment. The strain isolated in this study is labeled by a circle ●.

Table S1. Nucleotide and amino acid homology of S proteins of PEDV/SD/2020 isolates.

Isolates	Identity (%)																										
	1	2	3	4	5	6	7	8	9	10	11	12	13	14	15	16	17	18	19	20	21	22	23	24	25	26	27
SM98	*	96.5	96.0	96.7	99.6	96.3	99.1	98.1	93.4	93.7	93.6	94.1	93.8	93.7	93.7	93.6	93.5	93.9	93.6	94	95.4	95.4	95.4	95.4	95.5	95.2	93.6
SD-M	95.0	*	96.6	99.6	96.8	99.5	96.3	98.3	93.7	93.8	93.8	94.2	94.0	93.8	94.0	93.5	93.6	93.9	93.6	94.2	95.8	95.9	95.9	95.8	96.0	95.4	93.8
CH-S	95.2	96.0	*	96.8	96.3	96.3	95.8	97.4	93.5	93.8	93.7	94.1	93.9	93.7	93.8	93.4	93.5	93.7	93.5	94.0	95.6	95.7	95.7	95.7	95.7	95.4	93.7
JS2008	95.3	99.1	96.4	*	97.0	99.1	96.4	98.4	94.0	94.1	94.1	94.5	94.3	94.2	94.3	93.9	94.0	94.2	94.0	94.5	96.0	96.2	96.1	96.1	96.3	95.8	94.1
CV777	99.1	95.8	96.2	96.1	*	96.5	99.4	98.5	93.8	94.0	94.0	94.5	94.1	94.0	94.1	93.9	93.8	94.2	93.9	94.3	95.7	95.8	95.7	95.7	95.7	95.5	93.9
KC189944.1	95.1	99.6	95.9	99.1	95.9	*	95.9	97.9	93.2	93.4	93.4	93.8	93.5	93.4	93.5	93.1	93.2	93.5	93.2	93.7	95.4	95.5	95.5	95.4	95.5	95.0	93.7
LZC	98.1	94.9	95.2	95.2	99.1	94.9	*	97.9	93.3	93.5	93.5	93.9	93.6	93.5	93.5	93.4	93.3	93.7	93.4	93.8	95.2	95.3	95.2	95.2	95.2	95.0	93.4
Virulent DR13	97.0	97.8	97.0	98.0	98.0	97.8	97.0	*	94.7	95.0	94.9	95.3	95.1	94.9	95.0	94.7	94.7	95.0	94.7	95.2	96.9	97.0	97.0	96.9	97.1	96.6	94.8
JSX-2014	91.9	92.1	92.3	92.6	92.7	91.9	91.8	93.6	*	98.9	97.8	98.9	99.5	97.6	99.4	97.9	97.3	97.7	97.5	98.2	96.1	96.6	96.3	96.5	96.3	96.5	98.5
CH-ZMDZY-11	92.6	92.5	93.1	93.1	93.4	92.4	92.5	94.2	97.9	*	98.0	99.2	99.4	97.8	99.3	98.1	97.5	97.9	97.6	98.4	96.4	96.6	96.6	96.5	96.6	96.7	98.7
CH-FJZZ-9-2012	92.4	92.7	93.4	93.1	93.3	92.5	92.3	94.1	97.5	97.9	*	98.2	98.2	98.2	98.1	98.4	97.9	98.4	98.1	99.3	95.2	95.3	95.3	95.2	95.3	95.5	97.6
BJ-2011	93.0	93.0	93.5	93.6	93.8	92.8	92.9	94.6	98.5	99.1	98.5	*	99.4	98.2	99.3	98.4	97.8	98.3	98.0	98.7	96.3	96.4	96.4	96.3	96.4	96.6	98.7
IA1	92.9	93.0	93.5	93.6	93.8	92.9	92.8	94.7	98.8	99.1	98.7	99.6	*	98.0	99.8	98.3	97.7	98.1	97.8	98.6	96.6	97.0	96.7	96.9	96.7	96.9	98.8
GD-A	92.8	92.8	93.3	93.2	93.6	92.6	92.6	94.5	97.5	97.8	98.3	98.6	98.5	*	97.9	98.1	99.3	99.2	99.8	98.8	95.1	95.2	95.2	95.2	95.3	95.4	97.6
USA-IA-2013-49379	92.8	92.9	93.3	93.5	93.6	92.7	92.7	94.6	98.7	98.9	98.6	99.5	99.9	98.3	*	98.2	97.6	98.0	97.7	98.5	96.4	96.8	96.6	96.7	96.6	96.7	98.8
HBQHD1	92.2	92.3	93.0	92.8	93.1	92.1	92.1	93.8	97.5	98.0	98.3	98.6	98.7	97.8	98.6	*	97.7	98.2	97.9	98.9	95.3	95.4	95.4	95.3	95.4	95.6	97.8
CH-GDGZ-9-2012	92.6	92.5	93.0	92.9	93.3	92.3	92.3	94.2	96.6	97.1	97.6	97.8	97.8	98.7	97.6	97.0	*	98.7	99.1	98.4	95.0	95.1	95.0	95.0	95.1	95.2	97.3
AJ1102	93.0	93.0	93.6	93.6	93.7	92.9	92.8	94.6	97.5	98.1	98.7	98.9	98.7	99.4	98.6	98.1	98.6	*	99.1	99.0	95.2	95.3	95.2	95.2	95.2	95.4	97.5
JS-A	92.4	92.4	92.9	92.8	93.2	92.2	92.3	94.1	97.3	97.5	98.1	98.2	98.2	99.6	98.1	97.5	98.3	99.0	*	98.6	94.9	95.1	95.0	95.1	95.1	95.2	97.4
ZJCZ4	92.8	93.0	93.8	93.6	93.6	92.9	92.7	94.6	97.8	98.3	99.3	98.9	99.0	98.8	98.8	98.6	98.1	99.1	98.4	*	95.6	95.7	95.7	95.6	95.7	95.8	98.0
IT-A	95.1	95.7	95.7	96.2	96.0	95.6	95.0	97.4	94.9	95.5	94.9	95.9	96.0	94.9	95.9	95.0	94.6	95.1	94.6	95.3	*	99.3	99.6	99.2	99.6	98.8	96.4
OH851	95.4	95.8	96.0	96.3	96.2	95.7	95.2	97.7	95.2	95.7	95.1	96.2	96.4	95.1	96.2	95.2	94.7	95.4	94.8	95.5	99.0	*	99.5	99.8	99.4	98.9	96.4
GER-L00933-K22	95.2	95.8	95.8	96.3	96.0	95.7	95.1	97.5	95.1	95.7	95.1	96.2	96.2	95.1	96.1	95.2	94.6	95.4	94.8	95.5	99.4	99.4	*	99.4	99.8	98.9	96.5
USA-2014IL-20697	95.3	95.7	95.9	96.2	96.1	95.6	95.2	97.6	95.2	95.7	95.0	96.1	96.3	95.2	96.2	95.2	94.6	95.3	94.9	95.4	98.9	99.6	99.3	*	99.3	98.8	96.3
FR-001-2014	95.2	95.9	95.8	96.4	96.0	95.8	95.1	97.5	95.1	95.7	95.1	96.2	96.2	95.1	96.1	95.2	94.6	95.4	94.8	95.5	99.4	99.4	100.0	99.3	*	98.8	96.4
ZL29	95.2	95.3	95.7	96.0	96.0	95.2	95.1	97.2	95.1	97.2	95.4	96.0	95.4	96.5	96.4	95.5	95.0	95.7	95.1	95.8	98.5	98.9	98.8	98.8	98.8	*	96.5
PEDV-SD/2020	92.7	92.8	93.2	93.4	93.5	92.7	92.6	94.4	98.0	98.3	98.0	98.9	99.1	97.9	99.0	98.1	97.2	98.1	97.6	98.3	95.8	95.9	96.0	95.9	96.0	96.2	*

Note: S nucleotide sequence homology is shown in the gray background and S amino acid homology is shown in white backgroud
